# C-reactive protein for prediction of atrial fibrillation recurrence after catheter ablation

**DOI:** 10.1186/s12872-020-01711-x

**Published:** 2020-09-29

**Authors:** Pascal B. Meyre, Christian Sticherling, Florian Spies, Stefanie Aeschbacher, Steffen Blum, Gian Voellmin, Antonio Madaffari, David Conen, Stefan Osswald, Michael Kühne, Sven Knecht

**Affiliations:** 1 Division of Cardiology, Department of Medicine, University Hospital Basel, Petersgraben 4, 4031 Basel, Switzerland; 2 Cardiovascular Research Institute Basel, University Hospital Basel, Basel, Switzerland; 3 Population Health Research Institute, McMaster University, Hamilton, Ontario Canada

**Keywords:** Atrial fibrillation, Catheter ablation, C-reactive protein, Recurrence

## Abstract

**Background:**

Inflammation plays an important role in the initiation and progression of atrial fibrillation (AF), but data about the relationship between subclinical inflammation and recurrence of AF after catheter ablation remains poorly studied. We aimed to assess whether plasma levels of C-reactive protein (CRP) are associated with long-term AF recurrence following catheter ablation.

**Methods:**

Prior to the intervention, plasma CRP concentrations were measured in patients who underwent first catheter ablation for AF. AF recurrence was evaluated after 12 months and defined as any AF episode longer than 30 s recorded on either 12-lead electrocardiogram, 24-h Holter or 7-day Holter monitoring. Multivariable adjusted Cox models were constructed to examine the association of CRP levels and AF recurrence.

**Results:**

Of the 711 patients (mean age: 61 years, 25% women) included in this study, 247 patients (35%) experienced AF recurrence after ablation. Patients who were in the highest CRP quartile had a higher rate of recurrent AF compared to those who were in the lowest quartile (53.4 vs. 33.1% at 1 year of follow-up; *P* = 0.004). The adjusted hazard ratios (aHR) of recurrent AF across increasing quartiles of CRP were 1.0 (reference), 1.26 (95% confidence interval [CI], 0.86–1.84), 1.15 (95% CI, 0.78–1.70) and 1.60 (95% CI, 1.10–2.34) (P trend = 0.015). A similar effect was observed when CRP was analyzed as continuous variable (aHR per unit increase, 1.21; 95% CI, 1.05–1.39; *P* = 0.009). When a predefined CRP cut-off of 3 mg/l was applied, patients with CRP levels of 3 mg/l or above had a higher risk of AF recurrence than those with levels below (aHR, 1.44; 95% CI, 1.06–1.95; *P* = 0.019).

**Conclusions:**

Increasing pre-interventional CRP levels are associated with a higher risk of AF recurrence in patients undergoing catheter ablation for AF.

**Trail registration:**

ClinicalTrials.gov identifier, NCT03718364.

## Background

Atrial fibrillation (AF) currently affects more than 18 million Europeans and its prevalence is increasing rapidly [1, 2]. Patients with AF face a high risk of stroke, heart failure and death [3, 4]. Catheter ablation has become a well-established option for antiarrhythmic drug refractory AF, and it is accepted as first-line therapy in patients with symptomatic paroxysmal AF [5–7]. However, the rates of AF recurrence after an initially successful catheter ablation are high, with estimates ranging from 24 to 50% within the first months [8–10].

Epidemiologic and histological evidence suggests that inflammation may play an important role in the initiation and maintenance of AF [11, 12]. In the Women’s Health Study, elevated levels of pro-inflammatory biomarkers measured at baseline were associated with a higher risk of new-onset AF [13]. This and other studies showed that elevated levels of C-reactive protein (CRP), which is a well-known marker for local and systemic inflammation, are independently associated with an increased risk of incident AF [13–17]. Elevated baseline CRP levels predict AF recurrence following a successful electrical cardioversion [18], but the role of inflammation in the risk of AF recurrence after catheter ablation is less well-defined. Smaller and older studies with partially outdated interventional strategies showed inconsistent results [19–22]. Systemic inflammation may induce an electrical and mechanical substrate that then promotes AF episodes and increases the susceptibility for recurrent AF after ablation [11]. We therefore aimed to evaluate the association of elevated levels of CRP measured prior to catheter ablation with AF recurrence during a follow-up of 12 months.

## Methods

### Study design and population

The study population was derived from the Swiss Atrial Fibrillation Pulmonary Vein Isolation (SWISS-AF PVI) study, an ongoing prospective cohort study in Switzerland enrolling patients > 18 years of age with diagnosed AF who are scheduled to undergo first elective catheter ablation. Exclusion criteria were presence of longstanding persistent or permanent AF, and inability or unwillingness to participate in the study. Also, patients were excluded if they were experiencing acute inflammatory conditions (such as pneumonia or urosepsis) or showed symptoms of acute illness at baseline. However, this group of patients was eligible for enrolment 4 weeks after stabilization of their acute episode. None of the patients had diagnosed rheumatoid arthritis at baseline. The study protocol was approved by the local ethics committee, and written informed consent was obtained from all participants.

### Study procedures

Data on personal and medical characteristics were collected at baseline by study personnel using standardized case report forms. Information on demographics, lifestyle habits, medical history, and current medications was obtained. Prior to the ablation procedure, we performed transthoracic echocardiography to quantify left atrial dimension and left ventricular ejection fraction (LVEF). Type of AF was classified according to the current guidelines either as paroxysmal (self-terminating AF, within 48 h) or persistent AF (episodes lasting longer than 7 days, including episodes terminated by cardioversion after 7 days or more) [23].

### Blood sampling

Venous blood samples were obtained and processed at the day prior to ablation. Plasma levels of CRP were measured using an immunoturbidimetry on latex assay (C-Reactive Protein Gen.3, Roche Diagnostics). This assay allows quantification of CRP plasma levels below 2 mg/L (detection range of 0.3 to 350 mg/L), which is sufficient for cardiovascular risk assessment [24–26].

### Catheter ablation

Catheter ablation procedures aimed to restore sinus rhythm through isolation of all pulmonary veins. Ablation strategy and mapping system selection was left to the discretion of the electrophysiologist. Only experienced electrophysiologists who have had performed several AF ablation procedures, including pulmonary vein isolation, using the same approach were allowed to perform the ablation procedures. At the discretion of the operators, additional lesions were placed. The operators were unaware of the patients measured CRP levels.

### Follow-up and outcome

Regular follow-up visits in outpatient clinics were conducted by physicians at 3, 6, and 12 months. At each visit, a detailed medical and physical examination, 12-lead electrocardiogram, 24-h Holter, and 7-day Holter monitoring at 12 months were performed. In some patients the 7-day Holter was performed beyond the scheduled 12-month visit. All recordings were centrally collected at the University Hospital Basel and outcomes were adjudicated by trained study personnel and cardiologists. The outcome was AF recurrence defined as any AF or atrial tachycardia episode lasting longer than 30 s, which is in accordance with the 2017 Heart Rhythm Society expert consensus statement [27].

### Statistical analysis

A blanking period was applied to exclude events that occurred during the first 90 days after ablation, as recommended by the current guidelines [27]. Baseline characteristics were stratified according to quartiles of CRP levels. Continuous variables are described as mean ± standard deviation or as median and interquartile range (IQR) and categorical variables are presented as counts (percentages) and compared using tests for linear trend across CRP quartiles.

We used a multistage process to address the effect of CRP levels on the rates of AF recurrence that occurred after the blanking period. First, we divided patients into quartiles of CRP levels; the lowest quartile was defined as the reference. Incidence rates stratified by CRP quartiles were calculated at 1 year of follow-up and compared using the trend test for survival functions. Kaplan-Meier survival curves were used to estimate the cumulative incidence of recurrent AF across CRP quartiles and compared using log-rank test. We constructed Cox proportional hazard models to assess the risk of AF recurrence across CRP quartiles. The first model was unadjusted, the second model was adjusted for age and sex, and the third model was adjusted for age, sex, body mass index, AF type (paroxysmal vs. persistent), history of hypertension (yes vs. no), history of heart failure (yes vs. no), history of obstructive sleep apnea (yes vs. no), AF duration in years, and left atrial diameter (LAD). Second, we log-transformed CRP levels and repeated the analysis process using CRP as a continuous variable. Multivariable models were adjusted for the same set of variables as described above.

In additional analyses, we stratified the study population at a CRP cut-off value of 3 mg/l, a level known to be associated with high cardiovascular risk [28–30]. We evaluated whether the rates of AF recurrence differed in patients below or above this value and constructed the same multivariable models as described above.

All analyses were performed using Stata, version 13 (StataCorp. 2013. College Station, TX: StataCorp LP) and a two-sided *P*-value < 0.05 was considered to indicate statistical significance.

## Results

From April 29, 2010, through May 11, 2017, we enrolled 847 AF patients in the study. Of those, 748 patients completed at least one follow-up visit (Figure S1 in the Supplement). We excluded 37 patients due to missing CRP values at baseline (*n* = 33) and because blood samples were drawn after catheter ablation (*n* = 4). The median follow-up duration was 1.0 years (IQR, 0.5–1.2). Of the 711 AF patients included in our analyses, 247 (35%) experienced AF recurrence after ablation. Table 1 shows baseline characteristics of patients by quartiles of CRP levels. Significant differences were observed for age, sex, body-mass index, AF type, LAD and LVEF in the baseline echocardiogram, white blood cell count, for the frequency of hypertension and heart failure as well as for the history of amiodarone treatment across CRP categories.

**Table 1:** Baseline characteristics according to CRP level groups

	CRP Level				
Characteristic	Quartile 1 (***N*** = 201)	Quartile 2 (***N*** = 171)	Quartile 3 (***N*** = 167)	Quartile 4 (***N*** = 172)	P trend
Age, years	59.1 ± 10.7	60.1 ± 8.8	61.5 ± 8.8	61.6 ± 9.5	0.016
Female sex	42 (21)	36 (21)	45 (27)	50 (30)	0.019
Body mass index, kg/m^2^	25.4 ± 3.9	27.4 ± 4.0	27.7 ± 4.3	29.2 ± 6.1	< 0.001
Systolic blood pressure, mm Hg	135 ± 17	135 ± 18	135 ± 18	136 ± 20	0.78
Type of atrial fibrillation					< 0.001
Paroxysmal	139 (70)	108 (63)	97 (58)	90 (52)	
Persistent	61 (30)	63 (37)	69 (42)	82 (48)	
Atrial fibrillation duration, years	2.3 (0.6–6.6)	1.7 (0.5–4.8)	2.2 (0.6–6.1)	2.0 (0.6–5.6)	0.72
Echocardiographic parameters					
LAD, mm	39 ± 7	42 ± 7	42 ± 7	43 ± 6	< 0.001
LVEF, %	58 ± 10	57 ± 9	57 ± 10	55 ± 12	< 0.001
Laboratory data					
WBC, mm^3^	5860 (5020–6840)	6135 (5135–7515)	6440 (5290–7820)	6900 (5740–8400)	< 0.001
Serum creatinine, μmol/l	84 (74–95)	82 (74–93)	83 (72–94)	84 (73–97)	0.94
Medical history					
Coexistent atrial flutter	36 (18)	35 (20)	30 (18)	29 (17)	0.68
Hypertension	93 (47)	84 (50)	90 (54)	117 (69)	< 0.001
Diabetes	13 (7)	10 (6)	12 (7)	20 (12)	0.07
Stroke	20 (10)	9 (5)	10 (6)	15 (9)	0.64
Heart failure	11 (6)	11 (7)	16 (10)	26 (15)	0.001
Myocardial infarction	9 (5)	8 (5)	8 (5)	13 (8)	0.23
History of amiodarone treatment					< 0.001
Current	30 (15)	25 (15)	29 (17)	37 (22)	
Previous use	24 (12)	29 (17)	30 (18)	40 (23)	
Never use	147 (73)	117 (68)	108 (65)	95 (55)	

Data are presented as means ± standard deviations or medians (interquartile range), and counts (percentages)

*P* values compares quartiles of CRP levels

*Abbreviations*: *LAD* left atrial diameter, *LVEF* left ventricular ejection fraction, *WBC* white blood cell count

**Fig. 1:**
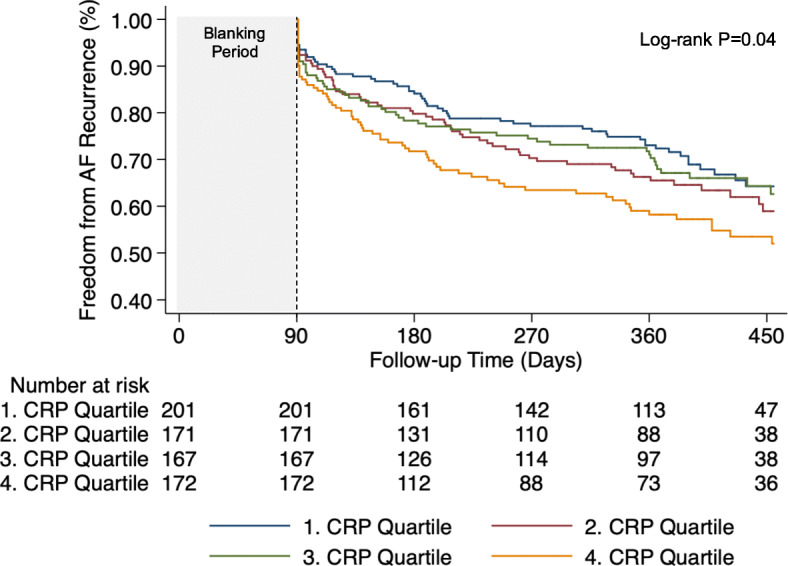
Kaplan-Meier curves for AF recurrence across quartiles of CRP levels

Table 2 presents the number of events and AF recurrence rates stratified by CRP quartiles. Incidence rates of AF recurrence for those in the second (0.8–1.4 mg/l), third (1.5–2.7 mg/l) and highest quartiles of CRP (> 2.7 mg/l) were higher as compared to those in the lowest quartile (< 0.8 mg/l). A threshold effect was observed between quartiles of CRP and AF recurrence, whereas the incidence rate was highest in the highest quartile (P for trend across quartiles = 0.017). Figure 1 shows the cumulative incidence of recurrent AF according to CRP quartiles after the blanking period (log-rank *P* = 0.04). In the fully adjusted model, the hazard ratios (aHR) for those in the second, third and highest quartiles of CRP, as compared to those in the in lowest quartile (reference), were 1.26 (95% confidence interval [CI], 0.86–1.84; *P* = 0.24), 1.15 (95% CI, 0.78–1.70; *P* = 0.48), and 1.60 (95% CI, 1.10–2.34; *P* = 0.014), respectively (Table 3). Data on CRP analyzed as continuous variable are shown in Table 3. A per unit increase in log CRP was associated with an aHR of 1.21 (95% CI, 1.05–1.39; *P* = 0.009).

**Table 2:** Incidence of recurrent AF after catheter ablation according to CRP quartiles

Variable	CRP level				P trend
	Quartile 1	Quartile 2	Quartile 3	Quartile 4	
Quartile value, mg/l	< 0.8	0.8–1.4	1.5–2.7	> 2.7	
Events/Patients	60/201	61/171	55/167	71/172	
Incidence, %^a^	33.1	41.9	37.0	53.4	0.017
95% CI	24.4–42.3	32.1–54.7	29.9–49.1	41.6–68.5	

*P* value was calculated by trend test of survival function across quartiles

^a^Incidence at 1 year of follow-up in percent

**Table 3:** Hazard ratios for recurrent AF after catheter ablation according to CRP quartiles and continuous CRP levels

Variable	CRP quartile				Continuous
	1^a^	2	3	4	Per unit increase in log CRP
Quartile value, mg/l	< 0.8	0.8–1.4	1.5–2.7	> 2.7	
Hazard ratio unadjusted	1.0	1.23	1.10	1.59	1.19
95% CI	–	0.86–1.76	0.78–1.62	1.13–2.25	1.04–1.35
*P* value	–	0.25	0.53	0.008	0.009
Hazard ratio adjusted for age and sex	1.0	1.24	1.09	1.54	1.17
95% CI	–	0.87–1.77	0.75–1.57	1.09–2.18	1.03–1.34
*P* value	–	0.23	0.65	0.015	0.018
Hazard ratio adjusted for age and sex + additional factors	1.0	1.26	1.15	1.60	1.21
95% CI	–	0.86–1.84	0.78–1.70	1.10–2.34	1.05–1.39
*P* value	–	0.24	0.48	0.014	0.009

Models adjusted for additional factors controlled for body mass index, AF type (paroxysmal vs. persistent), history of hypertension (yes vs. no), history of heart failure (yes vs. no), history of obstructive sleep apnea (yes vs. no), duration of AF (years), and LAD

Multivariable model included *n* = 659 patients

^a^CRP Quartile 1 served as reference group for each comparison

**Fig. 2:**
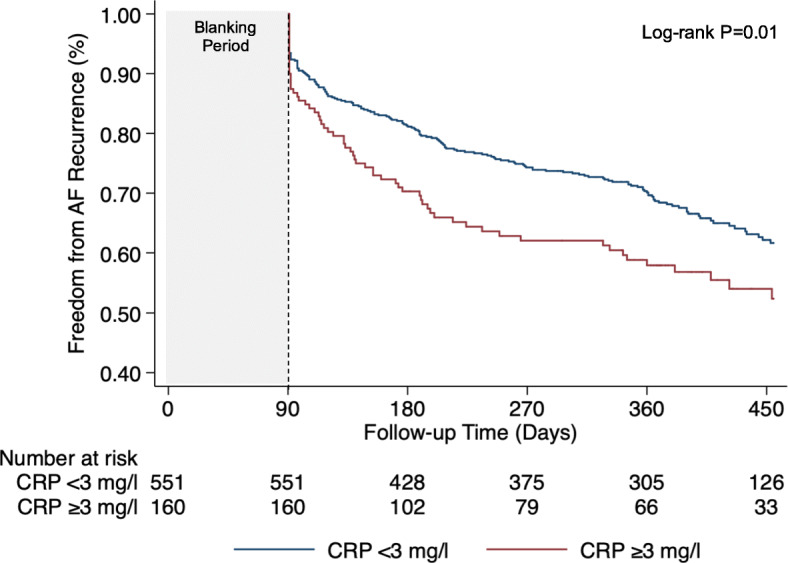
Kaplan-Meier curves for AF recurrence according to CRP levels above or below 3 mg/l

Patients who had CRP levels above the cut-off of 3 mg/l had higher rates of recurrent AF than patients with levels below 3 mg/l (66 patients [41%] vs. 181 patients [33%]; log-rank *P* = 0.009) (Table 4). Kaplan-Meier curves are shown in the Fig. 2. In multivariable analysis, the risk of AF recurrence was higher in patients who had CRP levels above 3 mg/l, as compared to those with levels below the cut-off value (aHR, 1.44; 95% CI, 1.06–1.95; *P* = 0.019) (Table 4).

**Table 4:** Hazard ratios for recurrent AF after catheter ablation according to CRP cut-off value of 3 mg/l

Variable	CRP level, mg/l	
	< 3^a^	≥ 3
Events/Patients	181/551	66/160
Incidence, %^b^	36.9	53.8
95% CI	31.6–43.2	41.6–69.6
Hazard ratio unadjusted	1.0	1.45
95% CI	–	1.09–1.92
*P* value	–	0.010
Hazard ratio adjusted for age and sex	1.0	1.41
95% CI	–	1.06–1.88
*P* value	–	0.017
Hazard ratio adjusted for age and sex + additional factors	1.0	1.44
95% CI	–	1.06–1.95
*P* value	–	0.019

Models adjusted for additional factors controlled for body mass index, AF type (paroxysmal vs. persistent), history of hypertension (yes vs. no), history of heart failure (yes vs. no), history of obstructive sleep apnea (yes vs. no), duration of AF (years), and LAD

Multivariable model included *n* = 659 patients

^a^CRP group < 3 mg/l served as reference for each comparison

^b^Incidence at 1 year of follow-up in percent

## Discussion

In this comprehensive analysis of AF patients who underwent first catheter ablation, increasing pre-interventional plasma levels of CRP were associated with a higher risk of AF recurrence. Patients in the highest CRP quartile had a 60% higher risk of recurrent AF compared to those who were in the lowest CRP quartile after adjustment for known risk factors for recurrence. These results were consistent when CRP levels were analyzed as a continuous variable or with a cut-off at 3 mg/l, suggesting that our findings are robust and not dependent on specific cut-off values. Adjusting for a broad set of potential confounders had little effect on the risk estimates, suggesting that residual confounding is less likely.

Although it is well-established that inflammation is independently associated with the development of AF [13–15], much less is known about whether inflammation increases the risk for AF recurrence after catheter ablation. Markers of inflammation including fibrosis, leukocyte infiltrates and oxidative damage have been found in atrial tissue samples of subjects with AF [12, 31, 32]. These inflammatory features may contribute to electrical and structural remodeling in atrial tissue and, thus, may promote recurrence of AF [33]. In support of this observation, previous studies have reported a potential association between elevated CRP levels and AF recurrence after catheter ablation [34, 35]. However, these mainly retrospective studies were small, had short follow-up duration, and used different cut-off levels for CRP. One retrospective cohort study involving a small and highly-selected sample of patients with AF used a cut-off value for abnormally high CRP of 5 mg/l, a value which was selected based on the laboratory assay used in the hospital [36]. Our study included significantly more patients with long-term observation and used a well-known CRP cut-off value for cardiovascular risk stratification, which allowed us to validate and confirm the findings from previous studies. We have now found a consistent association of CRP with AF recurrence in a large sample of well-characterized AF patients, which significantly contribute to the knowledge in the interrelationship between subclinical inflammation and AF recurrence after ablation.

Assessment of inflammatory biomarkers other than CRP may help to better understand the potential association between inflammation and recurrence of AF after catheter ablation. In smaller studies elevated levels of interleukin-2 (IL-2) and interleukin-6 (IL-6) were found to be significantly associated with recurrent AF after ablation [37, 38]. Another study showed that a IL-6 receptor genetic variant was associated with a higher risk of AF recurrence, emphasizing that the IL-6 pathway may affect the recurrence of AF [39]. Other markers of inflammation have been related to the risk of AF recurrence after catheter ablation, including matrix metalloproteinase-2 and tumor necrosis factor-α [40]. Taken together, these findings suggest that chronic inflammation may have an arrhythmogenic effect and therefore could at least in part explain failure of catheter ablation.

The findings of the current analysis may have several implications. First, given that failure rates of AF ablation remain significant, our results provide potential targets for therapeutic strategies to improve ablation success. Prospective epidemiologic studies demonstrated that obesity, smoking, high blood pressure and diabetes are independently associated with elevated levels of CRP and other inflammatory biomarkers [41–44]. This is in line with our observation showing that patients with higher CRP concentrations had a higher proportion of comorbid conditions, such as hypertension and higher body-mass index. Targeted therapies of those risk factors and lifestyle modifications have been shown to reduce CRP levels and improve maintenance of sinus rhythm in AF patients [45–47], suggesting that these interventions not only have an anti-inflammatory effect, but also reduce the burden of AF. Whether such risk factor and lifestyle interventions improve success rates of catheter ablation for AF by means of decreasing inflammation and thus CRP levels is currently unclear.

Second, previous trials have evaluated whether administration of anti-inflammatory therapy shortly after catheter ablation would reduce AF recurrence [48, 49]. Although the studies showed favorable results with regard to AF recurrence rates with anti-inflammatory therapy, interpretation of the findings is limited mainly by the small sample of participants, the short follow-up duration and the administration of different types of anti-inflammatory agents. Moreover, these studies did not specifically target patients with elevated CRP levels prior to ablation. Thus, there remains an unmet need for a large randomized controlled trial to evaluate the risks and benefits of anti-inflammatory therapy after ablation in AF patients. At least one ongoing trial is addressing this issue (IMPROVE-PVI ClinicalTrials.gov number, NCT04160117). From a mechanistic point of view, the association between pre-interventional subclinical inflammation and AF recurrence requires further investigations into the underlying biologic mechanism.

### Strengths and limitations

The strength of this study is the prospective design including a large sample of well-characterized AF patients. Furthermore, the large number of confirmed events, the availability of many covariates and the low rate of missing values allowed us to perform meaningful analyses. Nonetheless, this study has some potential limitations that require discussion. First, patient recruitment started in 2011 and ablation techniques may have changed over time, which could have impacted procedural success rates. Second, we measured CRP only at baseline and thus, could not evaluate the effects of changes in levels over time, or the effect of postinterventional CRP levels. Such measures may have provided a better overview about subclinical inflammatory state of the patients included in this study. Third, the study sample included patients who were recruited at a Swiss study center and the generalizability of the results to other populations is unclear. Forth, more than 10% of patients who had undergone initial catheter ablation were lost to follow-up (Figure S1 in the Supplement) and did not have an ECG recording regarding their rhythm status which could have affected the results. Lastly, follow-up screening for AF was performed at 3, 6, and 12 months using predefined methods. It is possible however that AF recurrence events may have been missed.

## Conclusions

Our data demonstrate that increasing CRP levels measured prior to catheter ablation were associated with a higher risk of AF recurrence. These findings support the hypothesis that inflammation may be involved in the recurrence of AF after catheter ablation.

## Supplementary information

**Additional file 1: Figure S1.** Flow diagram of patient selection and follow-up.

## Data Availability

The datasets generated and analysed during the current study are not publicly available because the patient consent forms, as approved by the responsible ethics committee, do not allow the data to be made publicly available, but are available from the corresponding author on reasonable request.

## Abbreviations

AF: Atrial fibrillation
aHR: Adjusted hazard ratio
CI: Confidence interval
CRP: C-reactive protein
LAD: Left atrial diameter
LVEF: Left ventricular ejection fraction
