# Supplementary material for: C-reactive protein for prediction of atrial fibrillation recurrence after catheter ablation
Source: BMC Cardiovasc Disord. 2020 Sep 29;20:427. doi: 10.1186/s12872-020-01711-x (PMC7526257; doi:10.1186/s12872-020-01711-x)
Supplement: Supplementary file 1 — Additional file 1: Figure S1. Flow diagram of patient selection and follow-up. [file 12872_2020_1711_MOESM1_ESM.docx]

**C-reactive protein for prediction of atrial fibrillation recurrence**

**after catheter ablation**

Pascal Meyre, Christian Sticherling, Florian Spies, Stefanie Aeschbacher, Steffen Blum, Gian Voellmin, Antonio Madaffari, David Conen, Stefan Osswald, Michael Kühne, Sven Knecht

**Supplemental material**

**Figure S1** Flow diagram of patient selection and follow-up

**Figure S1 Flow diagram of patient selection and follow-up**

**
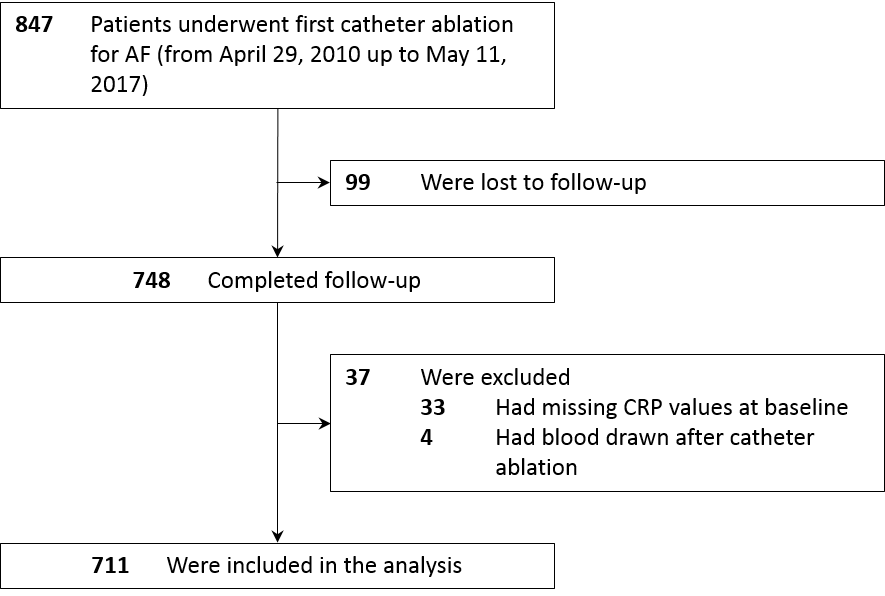
**
